# Increased neutrophils in inflammatory bowel disease accelerate the accumulation of amyloid plaques in the mouse model of Alzheimer’s disease

**DOI:** 10.1186/s41232-023-00257-7

**Published:** 2023-03-15

**Authors:** Ryusei Kaneko, Ako Matsui, Mahiro Watanabe, Yoshihiro Harada, Mitsuhiro Kanamori, Natsumi Awata, Mio Kawazoe, Tomoaki Takao, Yutaro Kobayashi, Chie Kikutake, Mikita Suyama, Takashi Saito, Takaomi C. Saido, Minako Ito

**Affiliations:** 1 Division of Allergy and Immunology, Medical Institute of Bioregulation, Kyushu University, 3-1-1 Maidashi, Higashi-ku, Fukuoka, 812-8582 Japan; 2 Division of Bioinformatics, Medical Institute of Bioregulation, Kyushu University, 3-1-1 Maidashi, Higashi-ku, Fukuoka, 812-8582 Japan; 3 Laboratory for Proteolytic Neuroscience, RIKEN Center for Brain Science (CBS), 2-1 Hirosawa, Wako-shi, Saitama, 351-0198 Japan; 4 Department of Neurocognitive Science, Institute of Brain Science, Nagoya City University Graduate School of Medical Sciences, Nagoya, Aichi 467-8601 Japan

**Keywords:** Alzheimer’s disease, Inflammatory bowel disease, Neutrophils

## Abstract

**Background:**

Alzheimer’s disease (AD) is one of the neurodegenerative diseases and characterized by the appearance and accumulation of amyloid-β (Aβ) aggregates and phosphorylated tau with aging. The aggregation of Aβ, which is the main component of senile plaques, is closely associated with disease progression. *App*^*NL-G-F*^ mice, a mouse model of AD, have three familial AD mutations in the amyloid-β precursor gene and exhibit age-dependent AD-like symptoms and pathology. Gut-brain interactions have attracted considerable attention and inflammatory bowel disease (IBD) has been associated with a higher risk of dementia, especially AD, in humans. However, the underlying mechanisms and the effects of intestinal inflammation on the brain in AD remain largely unknown. Therefore, we aimed to investigate the effects of intestinal inflammation on AD pathogenesis.

**Methods:**

Wild-type and *App*^*NL-G-F*^ mice at three months of age were fed with water containing 2% dextran sulfate sodium (DSS) to induce colitis. Immune cells in the brain were analyzed using single-cell RNA sequencing (scRNA-seq) analysis, and the aggregation of Aβ protein in the brain was analyzed via immunohistochemistry.

**Results:**

An increase in aggregated Aβ was observed in the brains of *App*^*NL-G-F*^ mice with acute intestinal inflammation. Detailed scRNA-seq analysis of immune cells in the brain showed that neutrophils in the brain increased after acute enteritis. Eliminating neutrophils by antibodies suppressed the accumulation of Aβ, which increased because of intestinal inflammation.

**Conclusion:**

These results suggest that neutrophils infiltrate the AD brain parenchyma when acute colitis occurs, and this infiltration is significantly related to disease progression. Therefore, we propose that neutrophil-targeted therapies could reduce Aβ accumulation observed in early AD and prevent the increased risk of AD due to colitis.

**Supplementary Information:**

The online version contains supplementary material available at 10.1186/s41232-023-00257-7.

## Background

Alzheimer’s disease (AD), the most common form of dementia, is a progressive age-related neurodegenerative disorder. The early stages of AD are characterized by amyloid-β (Aβ) production due to the abnormal cleavage of amyloid precursor protein (APP) by β- and γ-secretases, which oligomerize and aggregate into extracellular Aβ plaques called senile plaques. The disease is characterized by senile plaques, neurofibrillary tangles, and neuroinflammation, including microgliosis and astrogliosis [1]. These pathological features propagate throughout the brain as the disease progresses, resulting in synaptic loss, neuronal death, and, ultimately, cognitive decline [2].

Neurodegenerative diseases are closely related to the immune system [3], and the pathological impact of peripheral immune responses on AD has received considerable attention [4]. Peripheral immune cells, such as T cells, B cells, monocytes, and neutrophils, have been found in the brains of patients with AD and corresponding AD animal models. CD8^+^ T cells [5] and IFNγ^+^ CD4^+^ T cells [6] influence the pathogenesis of AD, and cytokines produced by immune cells alter the production and clearance of Aβ [6, 7]. Neutrophils that invade the brain in AD release neutrophil extracellular traps (NETs), which are DNA fibers studded with histones and myeloperoxidase, around Aβ plaques [8]. Thus, AD is closely related to peripheral immunity, but whether these immune cells improve or worsen AD remains controversial.

Interactions between the gut microbiota and brain have been demonstrated, and the molecular mechanisms are beginning to be elucidated. These interactions are called the gut-brain axes and closely related to many diseases [9]. Inflammatory bowel disease (IBD) causes inflammation in the intestine and is characterized by chronic symptoms with recurrent periods of flare-ups and remissions. IBD is reportedly caused by neuroinflammation, activation of the hypothalamic-pituitary-adrenal axis, changes in the blood-brain barrier, and gut microbiota imbalance [10]. It has long been suggested that there is an association between IBD, Crohn’s disease (CD), and cognitive impairment [11, 12]. A large, long-term cohort study from Taiwan reported that patients with IBD are at increased risk for dementia, particularly AD [13]. Recent study has shown that dextran sulfate sodium (DSS)-induced colitis enhances Aβ plaque accumulation in *App*^*NL-G-F*^ mice, which may result from the decrease in phagocytosis by microglia [14]. However, little is known about the relationship between AD and the systemic immune responses induced by IBD.

In this study, we investigated the effects of intestinal inflammation on the pathogenesis of AD. Colitis was induced in wild-type and *App*^*NL-G-F*^ mice, and immune cells in their brains were analyzed by scRNA-seq analysis. We found that acute colitis increased neutrophil infiltration into the brain, resulting in the accumulation of Aβ. Our results suggest that neutrophil-targeted therapies could reduce Aβ accumulation observed in early AD and prevent the increased risk of AD due to colitis.

## Materials and methods

### Animal models

*App*^*NL-G-F*^ mice (RBRC06344) were obtained from Dr. Takaomi C Saido and Dr. Takashi Saito at the RIKEN BioResource Center, Japan. These knock-in mice carry the humanized Aβ sequences with the addition of Swedish (NL), Arctic (G), and Iberian (F) mutations. This results in increased Aβ production as well as Aβ aggregation and increases the Aβ42/Aβ40 ratio [15, 16]. Wild-type (WT) C57BL/6J mice purchased from Jackson Laboratory Japan (Yokohama, Japan) and *App*^*NL-G-F*^ mice were maintained under appropriate conditions, including a 12-h light:12-h dark cycle and free access to food and water at Laboratory of Embryonic and Genetic Engineering, Medical Institute of Bioregulation, Kyushu University. Animal experiments were performed in strict accordance with the recommendations in the Guidelines for Proper Conduct of Animal Experiments of the Science Council of Japan. All experiments were approved by the Animal Research Committee and Ethics Committee of Kyushu University. Unless otherwise indicated, 3-month-old male and female C57BL/6J and *App*^*NL-G-F*^ mice were used. It is important to consider gender differences when studying AD, as epidemiological studies have shown that females have a higher prevalence and incidence of AD and that females have a greater Aβ plaque load in mouse models [17, 18]. However, the susceptibility to inflammation induced by DSS is higher in male mice, causing severe and aggressive disease [19]. Therefore, both male and female mice were used in this study.

### Antibodies and reagents

For flow cytometric analysis, fluorescein isothiocyanate (FITC), phycoerythrin (PE), peridinin chlorophyll protein-cyanine 5.5 (PerCP-Cy5.5), allophycocyanin (APC), PE-Cy7, APC-Cy7, Brilliant Violet (BV421), and BV711-conjugated antibodies were purchased from BD Biosciences, (San Jose, CA, USA), BioLegend (San Diego, CA, USA) or eBioscience (Thermo Fisher Scientific, Waltham, MA, USA). The following antibodies were used: anti-mouse CD45 (30-F11), anti-mouse CD45.2 (104), anti-mouse CD11b (M1/70), anti-mouse Gr1 (RB6-8C5), anti-mouse Ly6C (HK1.4), anti-mouse Ly6G (1A8). Fixable Viability Dye eFluor 780 (FVD780) (eBioscience) was used to remove dead cells.

For immunohistochemistry, the following antibodies were used: anti-human Aβ (1:200; 82E1, IBL), anti-mouse IBA1 (goat polyclonal antibody, 1:100; 011-27991, WAKO), anti-mouse GFAP (rabbit polyclonal antibody, 1:100 for cryosections, 1:500 for paraffin sections; G9269, Sigma), anti-mouse MRP8 (1:300; ab92331, abcam), Alexa Fluor 488-, 546-, or 633-conjugated goat anti-rabbit, mouse, or rat IgG (H+L, 1:300; Thermo Fisher Scientific), and Alexa Fluor 488-conjugated donkey anti-goat IgG (H+L, 1:300; Thermo). Anti-mouse Ly6G (1A8, BioXcell, Lebanon, NH, USA) (1 mg/kg), anti-rat kappa immunoglobulin light chain (MAR18.5, BioXcell) (1 mg/kg), and rat IgG2a isotype control (2A3, BioXcell) (2 mg/kg) were used as neutralizing antibodies. DNase I (Roche) (8 mg/kg), matrix metallopeptidase 9 (MMP-9) inhibitor II (Calbiochem) (10 mg/kg), and N-acetyl-l-cysteine (NAC) (Wako) (100 mg/kg) were used by intraperitoneal injection.

### DSS exposure

WT and *App*^*NL-G-F*^ mice were housed in cages according to experimental group. The control groups received only water, the acute colitis groups received DSS (2%, w/v, MW = 36–50 kDa; MP Biomedicals, LLS, Santa Ana, CA, USA) in their drinking water for one week, and the chronic colitis group received three cycles of alternating 2% DSS then only water, each for 5 days. To determine if colitis was induced, body weight was measured at the timing of the 2% DSS and drinking water exchanges and for one week prior to the day of mouse dissection. Weight loss was calculated as a ratio of the weight on each specific day over the weight on day 0. The photographs of the colons were taken, and the lengths were measured using ImageJ software (National Institutes of Health).

### Food allergy

Ovalbumin (OVA) (Sigma) were used as allergens. OVA was used as the food allergen only for the single-cell RNA sequencing experiments. WT and *App*^*NL-G-F*^ mice were intraperitoneally administered 0.1 mL of the allergen (0.5 mg/mL in PBS) mixed with 0.1 mL of Alum adjuvant. This was administered once per week for a total of three times. For 7 consecutive days after the above sensitization, 250 mg/mL of allergen was administered orally using a sonde at a dose of 0.2 mL per animal.

### Immunohistochemistry

Mice were anesthetized by inhalation with isoflurane and perfusion was performed by injecting PBS into the left ventricle followed by perfusion fixation via administration of 4% paraformaldehyde (PFA). After decapitation, the heads were immersed in 4% PFA and fixed at 4 °C for 24 h while the skin was cut and the skull was exposed. For frozen sections, the brains were removed and transferred in turn to 10%, 20%, and 30% sucrose solutions until completely submerged. The brains were then embedded in O.C.T. compound (Sakura Finetek) and rapidly frozen on dry ice and stored at – 20 °C. Frozen sections (10 μm) were prepared using a cryostat. For paraffin-embedded sections, coronal slices from the brain in cassettes were prepared by the Laboratory for Research Support, Medical Institute of Bioregulation, Kyushu University.

Brain cryosections and paraffin-embedded sections were treated on slides for 1 h at room temperature (25 °C) using Blocking One Histo (nacalai tesque). The sections were incubated with primary antibody in 5% BSA/PBS overnight at 4 °C, washed twice with PBS containing 0.1% Triton X-100 (PBS-T) and once with PBS, and incubated with the secondary antibody for 1 h at room temperature. The brain sections were sealed with cover glasses using a mounting agent. The images were taken with BZ-X700 (Keyence) and image analysis was performed using Keyence software.

### H&E staining

The cecum to distal colon was dissected and removed from the mice that had been perfused with PBS and stored in PBS. After fat and muscle were removed and photographs were taken side by side for each treatment group, the cecum was removed and washed internally with PBS. The colon was opened and rolled before immersion in 4% PFA. The Laboratory for Research Support of the Institute of Bioregulatory Medicine processed the tissue samples up to paraffin permeabilization. The samples were then paraffin-embedded and cut into 5 μm sections using a microtome, placed on glass slides, and then stretched using a paraffin stretching machine. The glass slides were placed in a staining basket and deparaffinized with xylene and ethanol. After staining with Meyer’s hematoxylin solution for 3 min and eosin solution for 1 min, they were dehydrated with ethanol and xylene and sealed with Paramount D (FALMA, Tokyo, Japan).

### ELISA

Mice were anesthetized by inhalation with isoflurane, blood in the heart was collected with a 26-G needle and 1 mL syringe and left at room temperature for 30 min. After centrifugation at 7000×*g* for 5 min the supernatant was collected and stored at − 80 °C. Enzyme-linked immunosorbent assay (ELISA) kits (Thermo Fisher Scientific) were then used according to the manufacturer’s instructions to measure IL-6 levels.

### Flow cytometry

Mice were anesthetized by inhalation with isoflurane, and blood from the hearts of mice was placed in heparin-coated tubes and treated with RBC lysis buffer. The following monoclonal antibodies were used for flow cytometric analysis at a dilution of 1/400: anti-CD45, anti-CD11b, anti-Ly6G, anti-Ly6C, anti-Ly6G/Ly6C (Gr-1) antibodies. For intracellular staining, after staining with antibodies against cell surface proteins, cells were washed three times with MACS buffer, fixed with eBioscience™ IC Fixation Buffer (Thermo Fisher Scientific), permeabilized with eBioscience™ Permeabilization Buffer, and then stained with an antibody against an intracellular marker. Cells not stained with FVD780 were categorized as live cells. Data were acquired with LSRFortessa (BD Biosciences, San Jose, CA, USA) and analyzed with FlowJo. The gating strategies of neutrophils (CD45^+^CD11b^+^Ly-6G^intra+^) are shown in Supplementary Figure S4.

### Single-cell RNA sequencing (scRNA-seq)

#### Cell isolation

Three-month-old WT and *App*^*NL-G-F*^ mice were subjected to acute enteritis, chronic enteritis, and food allergy treatments described above. The CD45-PE antibody was injected intravenously 3 min before perfusion to label the immune cells in the blood vessels. Mice were anesthetized by inhalation with isoflurane, and after PBS perfusion, the brain parenchyma was removed and homogenized in 2% FBS/RPMI solution containing glucose and HEPES [20]. After straining and centrifugation at 400×*g* for 5 min, the pellet was resuspended in 37% Percoll (Cytiva), cells were isolated by centrifugation with slow acceleration/deceleration 800×*g* for 20 min and resuspended with filtered 0.5% BSA/PBS. Staining was performed with anti-CD45.2 and anti-CD11b antibodies. Totalseq-Hashtag antibody (BioLegend) was incubated with each sample, and CD45.2^+^ cells were sorted by FACSMelody (BD Biosciences).

#### Library preparation and NGS

Libraries were prepared according to the protocol of the Chromuim Next GEM Single Cell 5' Reagent Kits v2 (Dual Index, 10x Genomics, Pleasanton, CA, USA) using a Chromium controller (10× Genomics). Briefly, GEMs were prepared by loading barcoded Single Cell VDJ 5' Gel Beads, master mix containing cells and RT enzymes, and Oil onto Chromium Next GEM Chip K. Barcoded cDNA was then generated from the GEM. This cDNA was amplified by PCR reaction to generate V(D) J, 5' Gene Expression and Feature Barcode libraries. Sequencing was then performed using Novaseq 6000 (Illumina, San Diego, CA, USA) at the Laboratory for Research Support of the Institute of Bioregulatory Medicine, Kyushu University.

#### Preprocessing

FASTQ files were generated from the NGS data by Cell Range (v6.1.2) (10× Genomics). Feature barcode matrices were created from the FASTQ data by Cell Ranger Multi (mouse genome; Genome Reference Consortium Mouse Build 38, mm10), and then converted to Seurat objects.

#### Data analysis

Analysis was performed using Seurat (v4.0.0) in r (v.4.1.3)  (R Foundation for Statistical Computing, Vienna, Austria). Seurat objects for each dataset were created with the CreateSeuratObject function (min.cells = 3, min.genes = 200). Cells with more than 5% of mitochondrial genes and cells with nFeature_RNA less than 200 were filtered out. Expression data were normalized with the normalizeData function (scale.factor = 10,000) and scaled with the ScaleData function. Highly variable genes in each dataset were identified using the FindVariableFeatures function. Principal component analysis (PCA) was performed on the identified highly variable genes using the RunPCA function. Enrichment of each PC was calculated using the JackStraw and ElbowPlot functions, and enriched PCs were selected for clustering and dimensionality reduction analysis. Clustering was performed using the FindClusters function (resolution = 0.5) and dimensionality reduction was performed using the RunUMAP function. Uniform manifold approximation and projection (UMAP) was used for dimensionality reduction and visualization. Statistically significant marker genes for each cluster were identified using the FindAllMarkers function (only.pos = TRUE, min.pct = 0.25, logfc.threshold = 0.25). Each identified cluster was then manually annotated with marker genes that have been previously reported as marker genes for defining cell subsets [21, 22]. Trajectory analysis of Seurat objects was performed using Monocle3 [23] package (v1.2.7) in R. Gene ontology (GO) analysis was performed using Metascape (v3.5) [24].

### Antibody and inhibitor treatment

This protocol was based on that from a previously reported study [25]. Three-month-old *App*^*NL-G-F*^ mice were treated with 2% DSS for 7 days and anti-Ly6G antibody and anti-rat kappa light chain antibody or isotype control were administered starting on day 3 of DSS treatment. Anti-Ly6G antibody (1 mg/kg) and anti-rat kappa immunoglobulin light chain (anti-rat κIgL antibody) (1 mg/kg), or rat IgG2a isotype control (2 mg/kg) were injected intraperitoneally. These antibodies were administered daily (anti-Ly6G and IgG2a isotype control) or every other day (anti-rat κIgL) over a 4-day period. For inhibitor treatment, DNase I (8 mg/kg), MMP-9 inhibitor II (10 mg/kg), and NAC (100 mg/kg) were administered intraperitoneally daily for 4 days starting on day 3 of DSS treatment.

### Statistics analyses

Data are expressed as mean ± s.e.m. Statistical significance was determined by one-way analysis of variance (ANOVA) followed by post hoc Tukey’s multiple-comparisons tests to analyze differences among three or more groups, and by unpaired Student’s *t* test or Mann-Whitney *U* test to analyze differences between two groups. *P* < 0.05 was considered to represent a significant difference (*P < 0.05; **P < 0.01; ***P < 0.001; ****P < 0.0001; ns not significant). Statistical analysis was performed using Prism 9.0 (GraphPad Software)

## Results

### Colon inflammation is induced by DSS and changes Aβ accumulation in the brains of App^NL-G-F^ mice

**Fig. 1:**
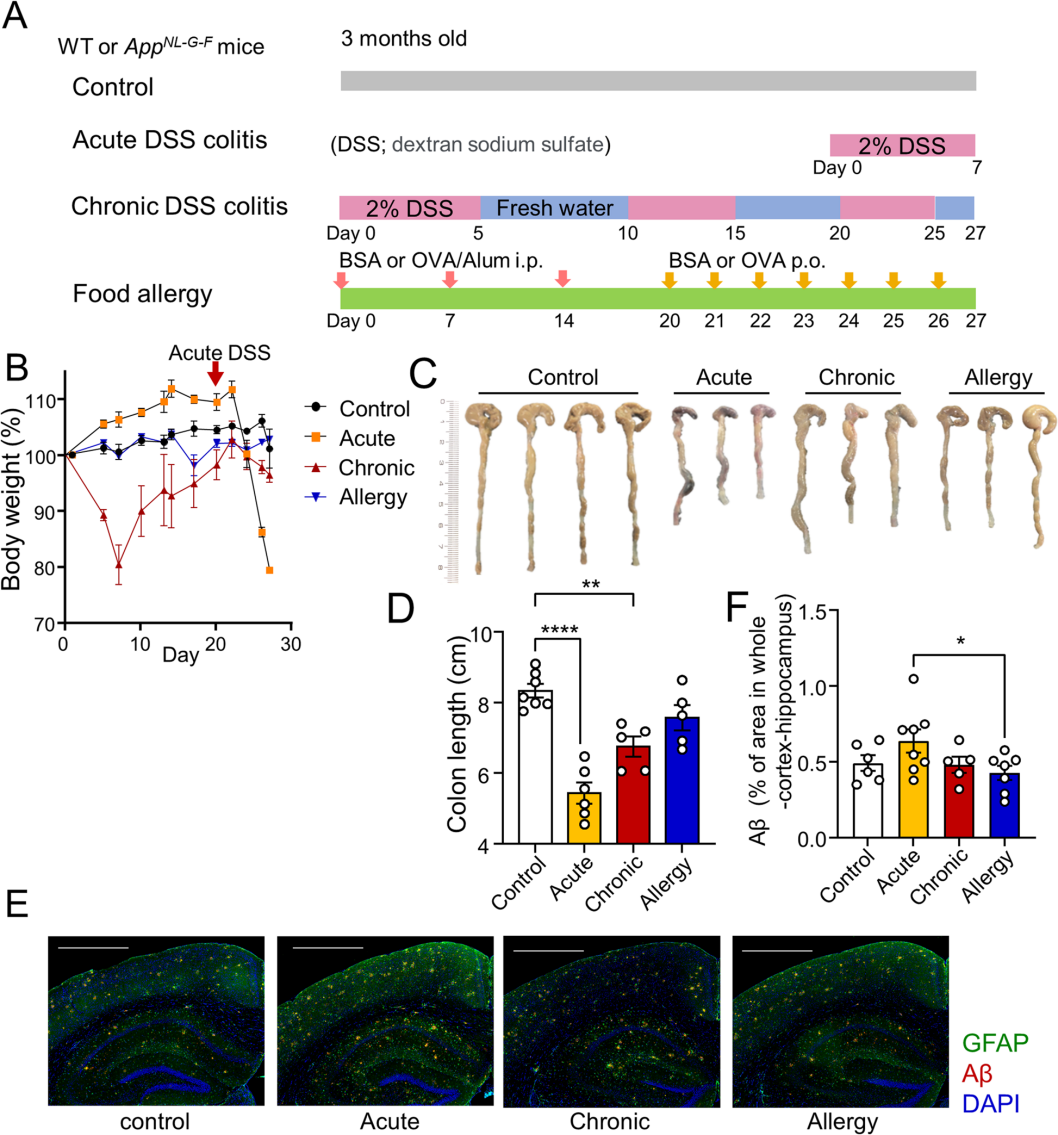
Changes of Aβ accumulation in the brains of *App*^*NL-G-F*^ mice with induced colon inflammation. **A** Schematic diagram of inducing acute DSS colitis, chronic DSS colitis, and food allergies. **B** Body weight of colitis-induced *App*^*NL-G-F* F^ mice. In acute colitis model, DSS treatment was started on day 20, indicated by the red arrow. **C**, D Images (**C**) and length (**D**) of colons from *App*^*NL-G-F*^ mice on day 27. **E**, **F** Immunohistochemical staining of glial cell activation and Aβ plaques. Representative images of Aβ, GFAP, and Iba1. Scale bar = 100 μm (**E**). The number of Aβ plaques per mm^2^ in cortex (**F**). Data points are individual mice from one representative experiment. *P* values were determined by one-way ANOVA (**D**, **F**). Data are shown as the mean ± s.e.m

To investigate the effects of intestinal inflammation on the pathogenesis of AD, we established acute and chronic models of intestinal inflammation induced by DSS administration and food allergy to ovalbumin (OVA) in 3-month-old *App*^*NL-G-F*^ and WT mice (Fig. 1A). Both WT and *App*^*NL-G-F*^ mice showed similar levels of weight loss after DSS treatment (Fig. 1B, Supplementary Figure S1A). As colitis is known to shorten the colon length, the large intestines were harvested from the mice, and their lengths were measured. Acute and chronic DSS colitis showed obvious shortening of the colon. (Fig. 1C, D). Histological staining of the colon showed severe inflammation and loss of crypts in both *App*^*NL-G-F*^ and WT mice in the acute colitis model and colon wall thickening and inflammation in the chronic colitis model (Supplementary Figure S1B, C). In addition, to assess IBD-like enteritis [26], IL-6 in the serum was measured by ELISA and was highly detected in the acute and chronic enteritis models but not in the control group or food allergy model (Supplementary Figure S1D).

To examine brain changes during acute colitis, chronic colitis, and food allergy, immunostaining for Aβ in the brain parenchyma of *App*^*NL-G-F*^ mice was performed. Compared to the control group, the number of Aβ plaques in the cortex was slightly higher in the acute colitis model, lower in the food allergy model, and comparable to that in the chronic colitis model (Fig. 1E, F). These data indicate that a systemic immune response to acute colitis can enhance Aβ accumulation in the brain.

### Neutrophils are increased in the brain of acute colitis-induced App^NL-G-F^ mice

**Fig. 2:**
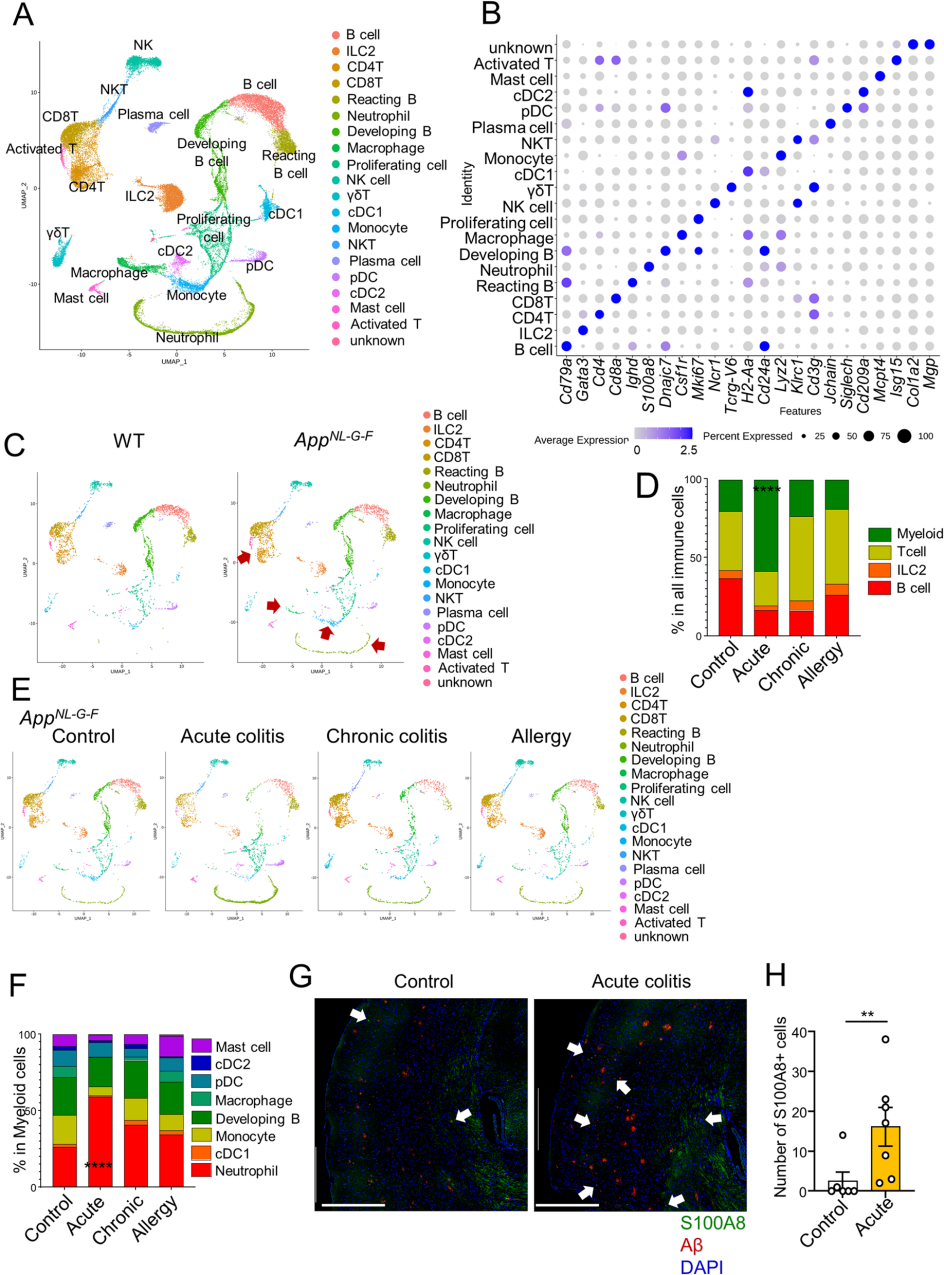
ScRNA-seq analysis of immune cells in the brain and increased neutrophils with acute colitis. **A** Uniform manifold approximation and projection (UMAP) visualization of scRNA-seq data of all samples. The immune cells in the brain were isolated by cell sorter and scRNA-seq analysis was performed. **B** Dot plots for selected differential expressed genes (DEG) and cell-type specific markers for the indicated clusters. **C** UMAP of control WT and *App*^*NL-G-F*^ mice. (WT, *n* = 4; *App*^*NL-G-F*^, *n* = 4) **D** Bar plot of percentage of myeloid cells, T cells, ILCs, B cells, in all immune cells in the brain. *P* value (control myeloid vs acute myeloid) was determined by Chi-square test. **E** UMAP of *App*^*NL-G-F*^ mice under different treatments. **F** Bar plot of indicated cells as percentage of myeloid cells in the brain. *P* value (control neutrophil vs acute neutrophil) was determined by Chi-square test. (D-F; *App*^*NL-G-F*^ Control, *n*= 4; Acute colitis, *n* = 4; Chronic colitis, *n* = 2; Allergy; *n* = 4). **G** Immunohistochemical staining of S100a8 (neutrophils) and Aβ plaque. Scale bar = 500 μm. White arrows indicated neutrophils. **H** The number of S100A8^+^ cells in the cerebral section. Data from single experiment (A-F). Representative images of three independent experiments (G, H). *P* values were determined by chi-square test (G), or two-tailed Mann-Whitney *U*-test (H). Data are shown as the mean ± s.e.m

To gain insight into the changes in immune cells in the brain induced by colitis, we induced colitis in WT and *App*^*NL-G-F*^ mice and performed a scRNA-seq analysis of CD45^+^ cells in the brain. Uniform manifold approximation and projection (UMAP) revealed the presence and heterogeneity of various immune cells (Fig. 2A). A plot of the marker gene characteristics of each cluster is shown in Fig. 2B. Compared to WT mice, *App*^*NL-G-F*^ mice showed a trend toward an increase in infiltrating immune cells, especially neutrophils, macrophages, monocytes, and T cells (Fig. 2C). These were roughly classified into four major subsets: T cells, B cells, myeloid cells, and ILC2 (Fig. 2D). A comparison of the percentages of these subsets by treatment group showed an increased percentage of myeloid lineage cells in the acute colitis model (Fig. 2D). Conversely, the percentage of T cells in the brain was increased in the chronic colitis and food allergy model mice compared to that in control mice (Fig. 2D, E). A more detailed analysis of myeloid cells revealed an increased proportion of neutrophils in the acute colitis model (Fig. 2E, F). Furthermore, immunostaining of the cortex confirmed an increased number of neutrophils in the brains of the acute colitis (Fig. 2G, H). Neutrophils accumulated only in areas of Aβ distribution, such as the cerebral cortex and hippocampus, but not in the cerebellum (data not shown). These data suggest that various immune cells are present in the brain, and that neutrophils are increased in the brain during acute colitis.

### Immature neutrophils are increased in App^NL-G-F^ mice with acute colitis

**Fig. 3:**
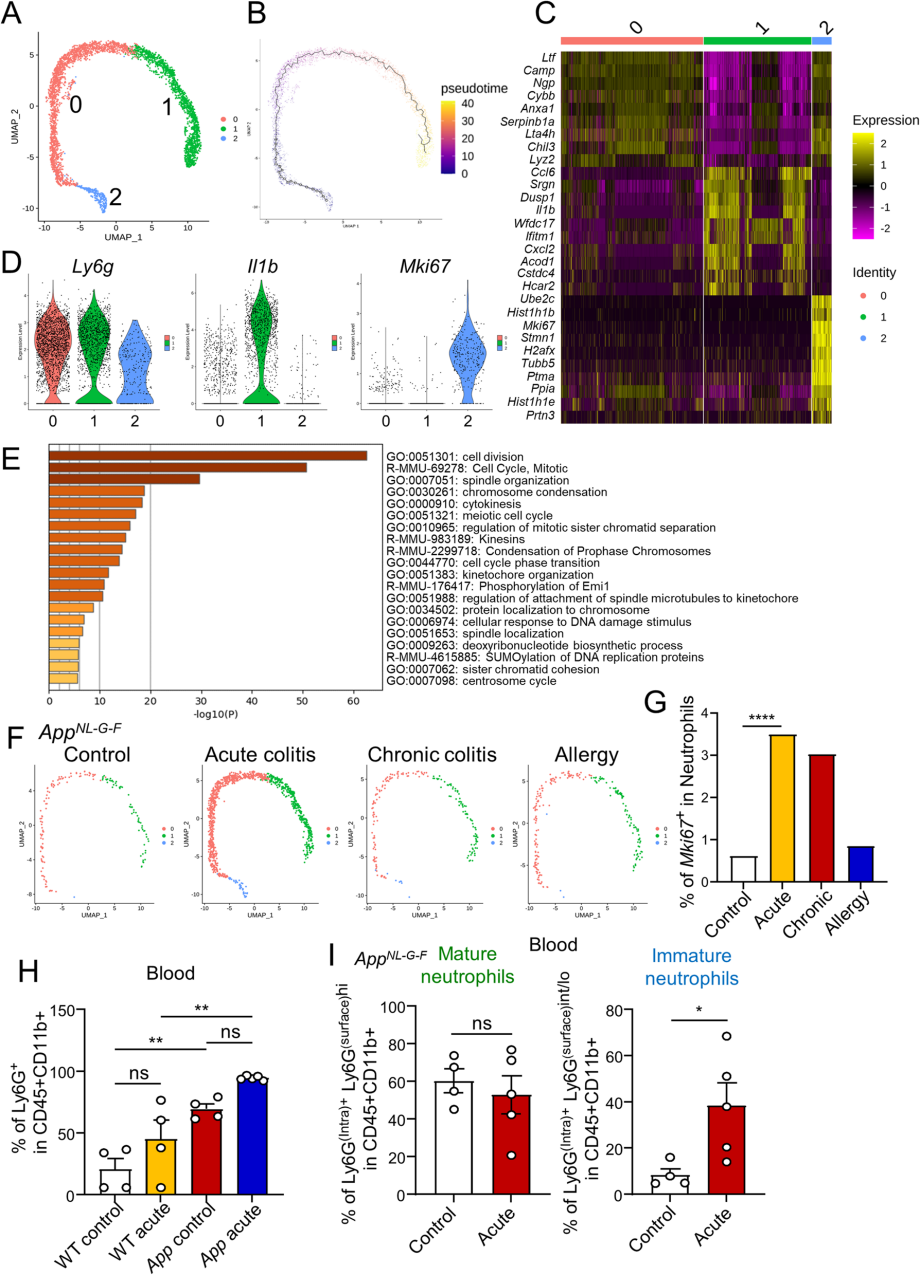
Increase in immature neutrophils in *App*^*NL-G-F*^ mice with acute colitis. **A** UMAP visualization of scRNA-seq data of neutrophils. **B** Developmental trajectory displayed on UMAP and colored by slingshot pseudotime. **C** Gene expression heatmap of the top 10 signature genes per cluster. **D** Violin plot of the expression levels of *Ly6G*, *Il1b*, and *Mki67* in the different cell clusters. **E** Gene Ontology (GO) analysis of DEGs of neutrophils in cluster2. **F** UMAP of neutrophils from *App*^*NL-G-F*^ mice under different treatments. **G** The percentage of *Mki67*^+^ cells in neutrophils in cluster 2. **H, I** Flow cytometric analysis of cells in the blood from WT or *App*^*NL-G-F*^ mice. The percentage of neutrophils in CD11b+ cells (**H**), and the percentage of neutrophils with high or intermediate/low expression of surface Ly6G (**I**). Data points are individual mice from one representative experiments. *P* values were determined by chi-square test (**G**), one-way ANOVA (**H**), or two-tailed Mann-Whitney *U* test (**I**). Data are shown as the mean ± s.e.m

Only neutrophil clusters were extracted from the scRNA-seq of immune cells in the brain for sub-clustering analysis. Data from scRNA-seq of neutrophils in the brain revealed three sub-clusters of neutrophils (Fig. 3A) and their sequential developmental trajectories (Fig. 3B). Neutrophils in cluster 2 expressed markers of cell cycle and immature neutrophils and showed high expression of *Mki67* and low expression of *Ly6g*. As indicated by the trajectory analysis, it was predicted that neutrophil differentiation would start from cluster 2 and differentiate into cluster 0, which is similar to the neutrophils in the bone marrow [27]. These neutrophils transitioned to express cluster 1 genes associated with mature neutrophils, such as *Il1b*, *Ccl*6, and *Ifitm1* (Fig. 3C, D). Gene ontology (GO) analysis of differential expressed genes (DEGs) neutrophils belonging to cluster 2 showed that genes involved in cell division (GO:0051301), mitotic spindle organization (GO:0007051), chromosome condensation (GO:0030261), and cytoplasmic division (GO:0000910) (Fig. 3E). We had found that the number of neutrophils was increased in acute colitis; however, there were no remarkable differences in the rate of neutrophils between treatments in clusters 0 or 1 (Fig. 3F). In contrast, the percentage of cluster 2 (*Mki67*^+^ neutrophils) was found to be increased in both the acute and chronic colitis models (Fig. 3G).

Flow cytometric analysis of blood neutrophils was performed in 3-month-old WT and *App*^*NL-G-F*^ mice treated with 2% DSS for 1 week to examine systemic neutrophil changes during colitis induction. The number of neutrophils in CD45^+^CD11b^+^ cells in the blood tended to increase in acute colitis (Fig. 3H). Newly produced immature neutrophils exhibit low Ly6G expression on the surface of the plasma membrane [8]. We defined neutrophils as intracellular Ly6G-positive cells and analyzed their surface Ly6G expression among them. The percentage of Ly6G^intra+^Ly6G^hi^ neutrophils (mature neutrophils) did not change, but the percentage of Ly6G^intra+^Ly6G^int/lo^ neutrophils (immature neutrophils) increased in *App*^*NL-G-F*^ mice with acute colitis (Fig. 3I). These data indicate that acute colitis increases neutrophils, especially immature neutrophils, in the brains and blood of *App*^*NL-G-F*^ mice.

### Neutrophil depletion reduces Aβ plaques in the brains of App^NL-G-F^ mice with acute colitis

**Fig. 4:**
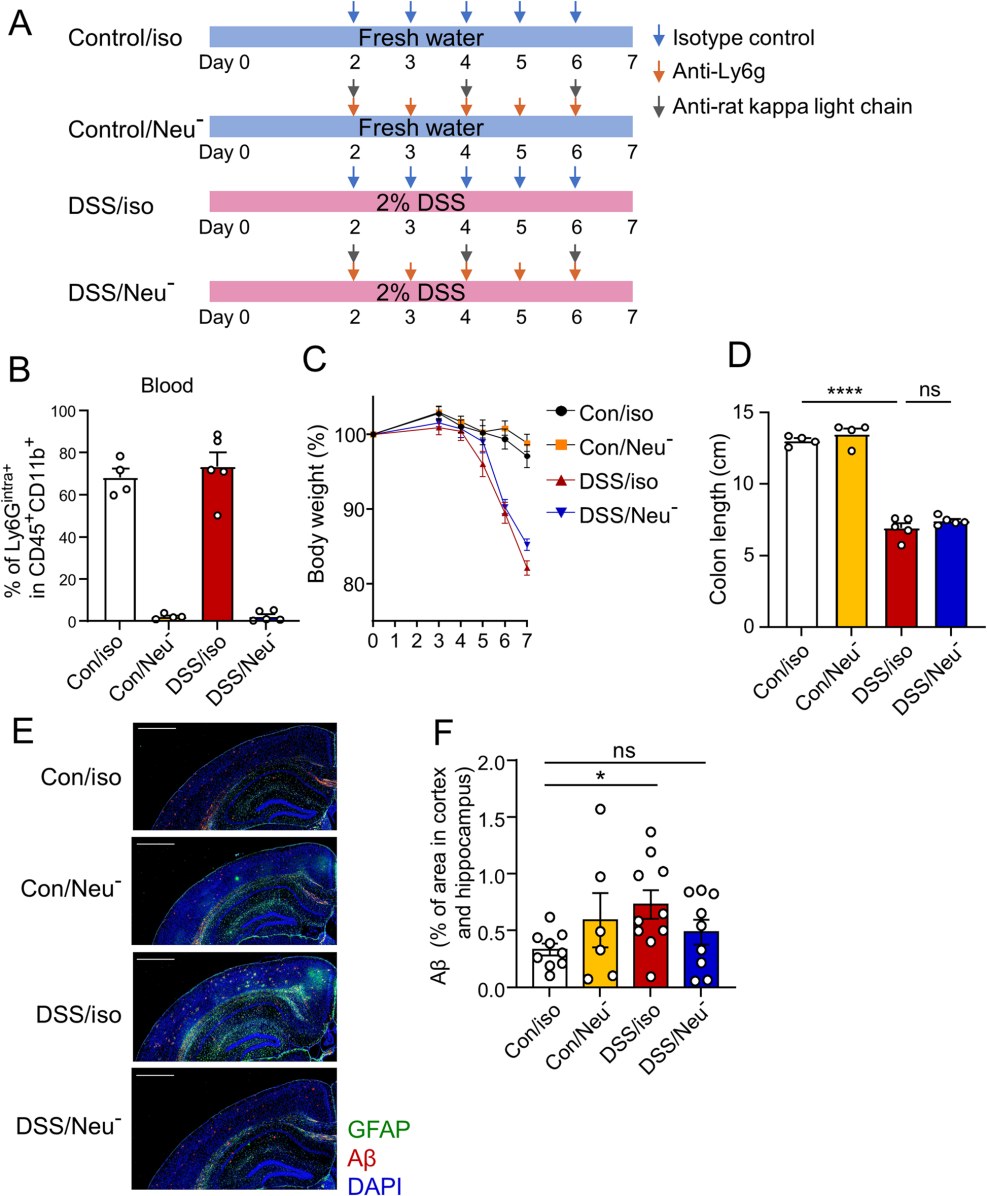
Reduction of Aβ plaques in the brains of *App*^*NL-G-F*^ mice with acute colitis by neutrophil depletion. **A** Schematic diagram of the experimental design to induce acute DSS colitis and deplete neutrophils. *App*^*NL-G-F*^ mice given water or 2% DSS were injected with anti-Ly6G antibody plus anti-rat kappa light chain antibody or isotype control antibody. **B** Flow cytometric analysis of cells in the blood. **C** The body weight of the mice. **D** The colon length of the mice. **E** Immunohistochemical staining of glial cell activation and Aβ plaques. Representative images of Aβ, and GFAP staining. Scale bar = 1 mm. **F** The percentage of Aβ plaque area in cortex and hippocampus. Data points are individual mice from one representative of two independent experiments (**B**–**E**), and in pooled data of two individual experiments (**F**). *P* values were determined by two-tailed Mann-Whitney *U* test (**D**, **F**). Data are shown as the mean ± s.e.m

Anti-Ly6G antibody, but not anti-Gr-1 antibody, was used to eliminate neutrophils without killing Ly6C-positive cells. Anti-Ly6G antibody is a rat IgG2a isotype that mediates neutrophil killing by Fc-dependent macrophage opsonization. The combination of anti-Ly6G and the kappa light chain of rat IgG2b activates both classical and alternative complement systems, resulting in rapid and efficient neutrophil elimination [25]. To confirm the role of neutrophils in the increased accumulation of Aβ during acute colitis, three-month-old *App*^*NL-G-F*^ mice were treated with 2% DSS for 7 days, and anti-Ly6G antibody and anti-rat κIgL antibody (neutrophil depletion: Neu^-^) or isotype control (isotype: iso) were administered starting on day 3 of DSS treatment (Fig. 4A). First, we confirmed that the neutrophil removal group showed a 97–98% reduction in neutrophils in the blood (Fig. 4B). DSS treatment resulted in similar levels of weight loss in both control and neutrophil-depleted groups (Fig. 4C). The length of the colon was significantly shorter in acute colitis, and neutrophil depletion did not affect the colon length (Fig. 4D, Supplementary Figure S2A). Acute colitis increased the accumulation of Aβ in the brain; however, this increase was reversed by depleting neutrophils at three months of age (Fig. 4E, F). A similar trend was observed in 12-month-old mice with acute colitis (Supplementary Figure S2B).

### Neutrophil-derived MMP-9 is involved in the accumulation of Aβ

**Fig. 5:**
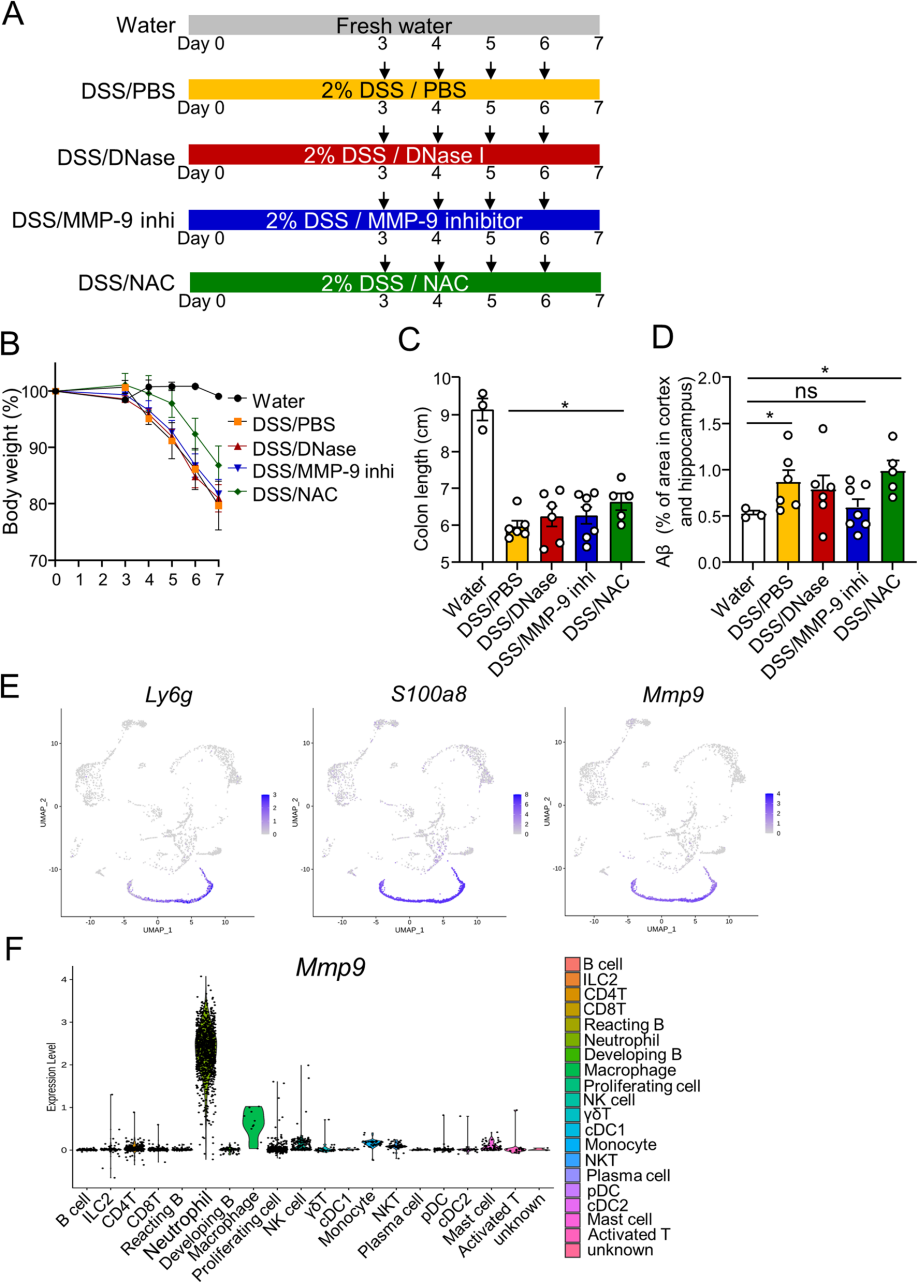
Involvement of MMP-9 in Aβ accumulation during acute colitis. **A** Schematic diagram of the experimental design to induce acute DSS colitis and administration of inhibitors. *App*^*NL-G-F*^ mice given water or 2% DSS were injected with DNase, MMP-9 inhibitor, or NAC. **B** The body weight of the mice. **C** The colon length of the mice. **D** The percentage of Aβ plaque area in cortex and hippocampus by immunohistochemical staining of Aβ plaques. **E** Feature plot of the expression levels of *Ly6g*, *A100a8*, and *Mmp9* in immune cells in the brain with acute colitis. **F** Violin plot of the expression of Mmp9 in the different cell clusters in the brain with acute colitis. Data points are individual mice in one experiment. *P* values were determined by two-tailed Mann-Whitney *U* test (**C**, **D**). Data are shown as the mean ± s.e.m

Neutrophils can release several inflammatory mediators, such as reactive oxygen species (ROS) and cytotoxic enzymes contained in granules including several matrix metalloproteinases (MMPs), which can cause tissue injury [28]. As MMP-9 expression correlates with the presence of neutrophils in models of brain injury [28], neutrophils are considered the main source of MMP-9. Furthermore, in transgenic models of AD, neutrophils have been reported to infiltrate and reside in areas of Aβ deposition in the brain, releasing NETs [8]. To identify factors involved in the accumulation of Aβ released from these neutrophils, we administered DNase, an inhibitor of NETs [29], MMP-9 inhibitor [30], or N-acetyl-l-cysteine (NAC, an inhibitor of ROS), to mice with DSS-induced colitis (Fig. 5A). The weight loss and shortened colon length with DSS administration were alleviated by ROS inhibition. However, no differences were observed with the other inhibitors (Fig. 5B, C). Under these conditions, the increased accumulation of Aβ in the brain caused by DSS colitis was suppressed by the MMP-9 inhibitor. Despite the alleviation of colitis by treatment with ROS inhibitor, Aβ accumulation was not reduced (Fig. 5D). Analysis of MMP-9-expressing cells from scRNA-seq data showed that neutrophils, which also expressed *Ly6g* and *S100a8*, were the immune cells with the highest MMP-9 expression in the brain during acute colitis, suggesting that neutrophils were the primary source of MMP-9 (Fig. 5E, F). *Mmp9* is expressed in all clusters, but the expression level of* Mmp9 *was particularly high in cluster 1, the mature neutrophils (Fig. S3). These results suggest that MMP-9 secreted by neutrophils may contribute to Aβ accumulation.

## Discussion

**Fig. 6:**
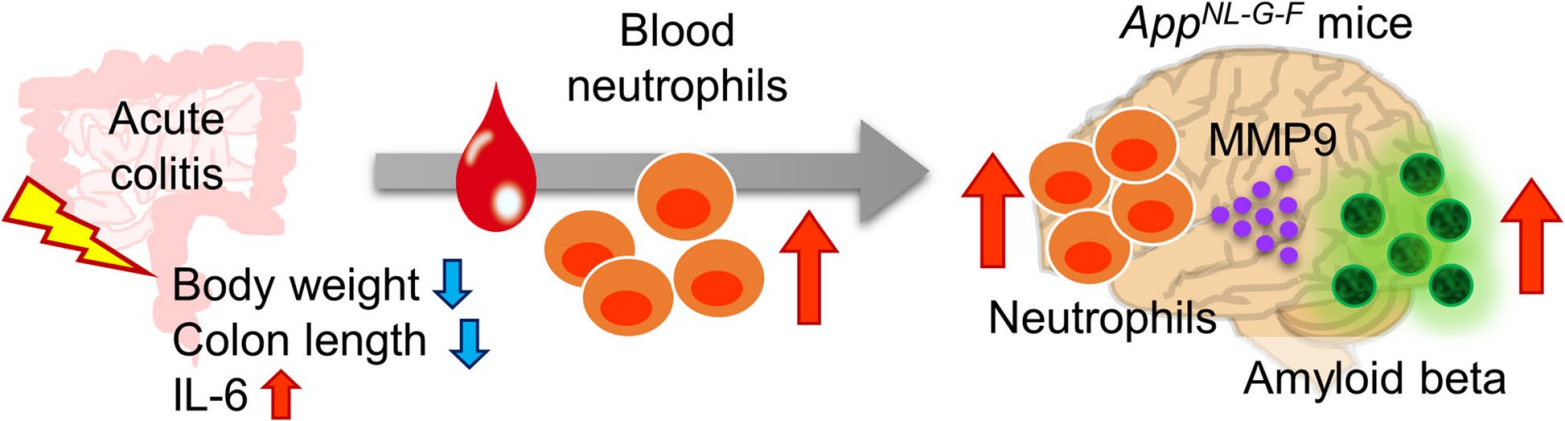
The schematic diagram of Aβ accumulation during acute colitis. Acute colitis induces weight loss, shortening of the colon and upregulation of inflammatory cytokines (such as IL-6). Neutrophils, especially immature neutrophils, increase in the blood from *App*^*NL-G-F*^ mice. The increase in neutrophils in the brain following an increase in the blood is associated with acute colitis-induced Aβ accumulation via increased MMP-9 activity in the brain

Three main pathways are assumed to transmit signals between the gut to the brain: the endocrine, immune, and nervous systems. Furthermore, the influence of the immune system can be divided into cellular and humoral factors by cytokines and other factors [9]. In this study, we identified a mechanism for immune system-mediated signaling from the gut to the brain during intestinal inflammation. Our data suggest a link between brain Aβ and the infiltration of immature neutrophils into the brain induced by colon inflammation in *App*^*NL-G-F*^ mice (Fig. 6). The impact of colitis on AD is supported by the following findings from our study: (1) Aβ accumulation in the brain is exacerbated by acute colitis, unchanged by chronic colitis, and tends to decrease with food allergy-induced enteritis; (2) acute colitis alters the percentage and number of immune cells in the brains of AD mice, particularly by increasing neutrophils in the blood and brain; and (3) removal of neutrophils and inhibition of MMP-9 suppressed the accumulation of Aβ in the brains of AD mice induced by acute colitis. Collectively, these data suggest that acute colitis increases neutrophils in the blood followed by increased neutrophils in the brain resulting in increased MMP-9 activity and Aβ accumulation.

Our insights into DSS-induced colitis and Aβ accumulation are supported by a recent study that showed that DSS can induce a moderate colitis in *App*^*NL-G-F*^ mice, resulting in increased Aβ accumulation with decreased microglial motility and phagocytosis [14]. Since our acute colitis model did not confirm the decreased CD68 expression shown by Sohrabi et al [14], different mechanisms may be involved rather than the phagocytic capacity of microglia. Since the one of the aims of this study is to examine the effects of systemic inflammation on the early accumulation of Aβ, we utilized 3-month-old mice, which correspond to the late 20s and 30s in humans. The peak onset of ulcerative colitis is known to be in the 20s, and the age of onset is earlier in familial Alzheimer's disease (40~50s) and Aβ accumulation is assumed to begin before onset [31]. A chronic colitis model is used to mimic the pathogenesis of human ulcerative colitis, but unexpectedly an increase in Aβ accumulation is found only in the acute colitis, not the chronic model. Previous reports show that peripherally inflammatory stimuli induce acute immune training and tolerance in the brain and epigenetic reprogramming of brain microglia [32]. In a mouse model of Alzheimer's disease, immune training with a single dose of LPS exacerbates amyloid-beta accumulation in the brain, while immune tolerance with continuous intraperitoneal administration of LPS alleviates it [32]. We speculate that the once increased Aβ decrease by the similar mechanism in our chronic colitis. For allergic models and AD, intracerebral injection of interleukin-4/13 ameliorates cognitive deficits in AD mice by inhibiting Aβ accumulation through increased Aβ clearance by M2-like activation of microglia [33]. Microglia treated with interleukin-4 may play a protective role against AD by increasing the uptake and degradation of Aβ in a process partially mediated by autophagy [34].

Previous studies have also shown that neutrophil depletion via the administration of Ly6G or Gr-1 antibodies in the 3xTg-AD mouse model improves cognitive symptoms, increases soluble Aβ, and reduces microgliosis compared to WT mice [8]. Another study, using a microfluidic platform, demonstrated that neutrophils activated by microglia can cross the blood-brain barrier and interact with Aβ-reactive microglia, resulting in the production of inflammatory cytokines [35]. Although this group showed that neutrophils stimulation by microglia increases neutrophil motility [35], the factors that activated neutrophils in our study are unclear.

The origin of neutrophils that infiltrate the brain during acute colitis is unclear. It is possible that humoral factors, such as granulocyte-colony stimulating factor, stimulate the production of neutrophils in bone marrow which then migrate to the brain via the blood. Neutrophils in the dura mater have also been reported to originate from the bone marrow in the skull [22] and also could be the source of the increased number of neutrophils in the brain due to acute colitis. Mouse models of AD have been shown to have more neutrophils in the brain, even in the absence of intestinal inflammation [8]. Distinguishing between these neutrophils and neutrophils proliferated by acute intestinal inflammation will help to elucidate the mechanism in more detail.

Neutrophil removal by anti-Ly6G antibody administration does not deplete newly generated neutrophils of bone marrow origin [25], and anti-Gr-1 antibody administration also recognizes Ly6C and may remove monocytes and macrophages [36]. In a previous study, it was shown that neutrophils newly generated after anti-Ly6G antibody administration had low expression of surface Ly6G. However, these could also be removed by dual-antibody neutrophil depletion [25]. Studies using Ly6G antibodies have also demonstrated that neutrophil adhesion in brain capillaries reduces cortical blood flow and memory function in a mouse model of Alzheimer's disease but does not affect Aβ accumulation [37]. To clarify the mechanism by which neutrophils increase Aβ accumulation, it is necessary to manipulate neutrophils specifically in the brain, rather than systemic treatments like the method used in our study.

Although increased Aβ accumulation by neutrophils has been reported to be caused by neutrophil-derived NETs [8], removal of NETs by DNase had no inhibitory effect on Aβ accumulation in our experiments. Alternatively, we found that MMP-9 inhibition suppressed Aβ accumulation. Several studies have reported the relationship between MMPs and AD. While MMP-2 has been expressed in neurons under steady state, MMP-9 is not but it is highly expressed in astrocytes in 2- to 6-month-old AD mouse models [38]. In situ experiments have shown that MMP-9 can degrade compact plaques of Aβ fibrils [39]. In addition, the increase in steady-state soluble Aβ in MMP-9 KO mice indicates that MMP-9 contributes to the degradation of small Aβ aggregates [40]. However, for insoluble Aβ, no change or a trend toward a decrease was observed with MMP-9 KO, suggesting that MMP-9 may promote the formation of large Aβ plaques. Overexpression of MMPs in HEK cells carrying the human APP Swedish mutation strongly increases the levels of β-secretase-derived C-terminal APP fragment and Aβ levels [38]. In our experiment, we systemically administered MMP-9 inhibitors, which also may inhibit astrocyte-derived MMP-9; therefore, the neutrophil-specific deletion of MMP-9 would be interesting.

Since this study only examined effects via the immune system, future studies should also include endocrine and nervous system-mediated signals between the gut and the brain to further understand the mechanisms in the crosstalk between AD and colitis. In addition, examining the contribution of neutrophils to Aβ plaque formation by specifically removing neutrophils from the brain is critical to understanding the role of neutrophils in enteritis and AD, as well as developing future therapeutic strategies targeting them.

## Conclusion

These results suggest that neutrophils infiltrate the brain parenchyma of AD during acute colitis and that neutrophil infiltration is significantly related to the progression of the disease. We also propose a strategy whereby neutrophil-targeted therapies could reduce Aβ accumulation observed in early AD and prevent the increased risk of AD due to colitis.

## Supplementary Information

**Additional file 1: Supplementary Figure S1.** The confirmation of induction of colitis. A Body weight of colitis-induced WT mice. In acute colitis model, DSS treatment was started on day 20, indicated by the red arrow. B, C H&E staining of colon from WT (B) or *App*^*NL-G-F*^ mice (C) induced acute DSS colitis, chronic DSS colitis, and food allergies. D The protein levels of IL-6 in the serum of *App*^*NL-G-F*^ mice with colitis. Data points are individual mice from one representative of two independent experiments (A, D). *P* values were determined by one-way ANOVA. Data are shown as the mean ± s.e.m. **Supplementary Figure S2.** Reduction of Aβ plaques in the brains of *App*^*NL-G-F*^ mice with acute colitis by neutrophil depletion. *App*^*NL-G-F*^ mice drinking with fresh water or 2% DSS water were injected with anti-Ly6G antibody plus anti-rat kappa light chain antibody or isotype control antibody. A The photos of colon of *App*^*NL-G-F*^ mice on day 7. B The percentage of Aβ plaques area in cortex and hippocampus by immunohistochemical staining. Data points are individual mice in one experiment. **Supplementary Fig. S3.** Cluster-specific gene expression in neutrophils. Dot plots for selected differential expressed genes (DEG) and cell-type specific markers for the indicated clusters. **Supplementary Figure S4.** Gating strategy for neutrophils in the blood. Gating strategy to analyze young and adult neutrophils in the blood by flow cytometry analysis as shown in Fig. 3 H-I, and Fig. 4B.

## Data Availability

Single cell RNA sequencing data have been deposited in the Gene Expression Omnibs of NCBI under accession no. GSE214747.

## Abbreviations

AD: Alzheimer’s disease
APC: Allophycocyanin
APP: Amyloid precursor protein
Aβ: Amyloid-β
BSA: Bovine serum albumin
CD: Crohn’s disease
DSS: Dextran sulfate sodium
FITC: Fluorescein isothiocyanate
FVD780: Fixable Viability Dye eFluor 780
IBD: Inflammatory bowel disease
MMP: Matrix metalloproteinase
NAC: N-Acetyl-L-cysteine
NETs: Neutrophil extracellular traps
OVA: Ovalbumin
PCA: Principal component analysis
PE: Phycoerythrin
PerCP-Cy5.5: Peridinin chlorophyll protein-cyanine 5.5
ROS: Reactive oxygen species
scRNA-seq: Single-cell RNA sequencing
UMAP: Uniform manifold approximation and projection

## References

[1] Long JM, Holtzman DM (2019). Alzheimer disease: an update on pathobiology and treatment strategies. Cell.

[2] Newcombe EA, Camats-Perna J, Silva ML, Valmas N, Huat TJ, Medeiros R (2018). Inflammation: the link between comorbidities, genetics, and Alzheimer's disease. J Neuroinflammation.

[3] Matheoud D, Sugiura A, Bellemare-Pelletier A, Laplante A, Rondeau C, Chemali M, Fazel A, Bergeron JJ, Trudeau LE, Burelle Y (2016). Parkinson's disease-related proteins PINK1 and Parkin repress mitochondrial antigen presentation. cell.

[4] Xie J, Van Hoecke L, Vandenbroucke RE (2021). The impact of systemic inflammation on Alzheimer's disease pathology. Front Immunol.

[5] Gate D, Saligrama N, Leventhal O, Yang AC, Unger MS, Middeldorp J, Chen K, Lehallier B, Channappa D, De Los Santos MB (2020). Clonally expanded CD8 T cells patrol the cerebrospinal fluid in Alzheimer's disease. Nature.

[6] Browne TC, McQuillan K, McManus RM, O'Reilly JA, Mills KH, Lynch MA (2013). IFN-γ Production by amyloid β-specific Th1 cells promotes microglial activation and increases plaque burden in a mouse model of Alzheimer's disease. J Immunol.

[7] Yamamoto M, Kiyota T, Horiba M, Buescher JL, Walsh SM, Gendelman HE, Ikezu T (2007). Interferon-gamma and tumor necrosis factor-alpha regulate amyloid-beta plaque deposition and beta-secretase expression in Swedish mutant APP transgenic mice. Am J Pathol.

[8] Zenaro E, Pietronigro E, Della Bianca V, Piacentino G, Marongiu L, Budui S, Turano E, Rossi B, Angiari S, Dusi S (2015). Neutrophils promote Alzheimer's disease-like pathology and cognitive decline via LFA-1 integrin. Nat Med.

[9] Carabotti M, Scirocco A, Maselli MA, Severi C (2015). The gut-brain axis: interactions between enteric microbiota, central and enteric nervous systems. Ann Gastroenterol.

[10] Maes M, Berk M, Goehler L, Song C, Anderson G, Gałecki P, Leonard B (2012). Depression and sickness behavior are Janus-faced responses to shared inflammatory pathways. BMC Med.

[11] van Langenberg DR, Yelland GW, Robinson SR, Gibson PR (2017). Cognitive impairment in Crohn's disease is associated with systemic inflammation, symptom burden and sleep disturbance. United European Gastroenterol J.

[12] Navabi S, Gorrepati VS, Yadav S, Chintanaboina J, Maher S, Demuth P, Stern B, Stuart A, Tinsley A, Clarke K (2018). Influences and impact of anxiety and depression in the setting of inflammatory bowel disease. Inflamm Bowel Dis.

[13] Zhang B, Wang HE, Bai YM, Tsai SJ, Su TP, Chen TJ, Wang YP, Chen MH (2021). Inflammatory bowel disease is associated with higher dementia risk: a nationwide longitudinal study. Gut.

[14] Sohrabi M, Pecoraro HL, Combs CK (2021). Gut inflammation induced by dextran sulfate sodium exacerbates amyloid-β plaque deposition in the AppNL-G-F mouse model of Alzheimer's disease. J Alzheimers Dis.

[15] Saito T, Matsuba Y, Mihira N, Takano J, Nilsson P, Itohara S, Iwata N, Saido TC (2014). Single App knock-in mouse models of Alzheimer's disease. Nat Neurosci.

[16] Sasaguri H, Nilsson P, Hashimoto S, Nagata K, Saito T, De Strooper B, Hardy J, Vassar R, Winblad B, Saido TC (2017). APP mouse models for Alzheimer's disease preclinical studies. EMBO J.

[17] Mielke MM, Vemuri P, Rocca WA (2014). Clinical epidemiology of Alzheimer's disease: assessing sex and gender differences. Clin Epidemiol.

[18] Mifflin MA, Winslow W, Surendra L, Tallino S, Vural A, Velazquez R (2021). Sex differences in the IntelliCage and the Morris water maze in the APP/PS1 mouse model of amyloidosis. Neurobiol Aging.

[19] Chassaing B, Aitken JD, Malleshappa M, Vijay-Kumar M (2014). Dextran sulfate sodium (DSS)-induced colitis in mice. Curr Protoc Immunol.

[20] Masuda T, Amann L, Sankowski R, Staszewski O, Lenz M, Errico P, Snaidero N, Costa Jordão MJ, Böttcher C, Kierdorf K (2020). Novel Hexb-based tools for studying microglia in the CNS. Nat Immunol.

[21] Brioschi S, Wang WL, Peng V, Wang M, Shchukina I, Greenberg ZJ, Bando JK, Jaeger N, Czepielewski RS, Swain A (2021). Heterogeneity of meningeal B cells reveals a lymphopoietic niche at the CNS borders. Science.

[22] Cugurra A, Mamuladze T, Rustenhoven J, Dykstra T, Beroshvili G, Greenberg ZJ, Baker W, Papadopoulos Z, Drieu A, Blackburn S (2021). Skull and vertebral bone marrow are myeloid cell reservoirs for the meninges and CNS parenchyma. Science.

[23] Cao J, Spielmann M, Qiu X, Huang X, Ibrahim DM, Hill AJ, Zhang F, Mundlos S, Christiansen L, Steemers FJ (2019). The single-cell transcriptional landscape of mammalian organogenesis. Nature.

[24] Zhou Y, Zhou B, Pache L, Chang M, Khodabakhshi AH, Tanaseichuk O, Benner C, Chanda SK (2019). Metascape provides a biologist-oriented resource for the analysis of systems-level datasets. Nat Commun.

[25] Boivin G, Faget J, Ancey PB, Gkasti A, Mussard J, Engblom C, Pfirschke C, Contat C, Pascual J, Vazquez J (2020). Durable and controlled depletion of neutrophils in mice. Nat Commun.

[26] Mudter J, Neurath MF (2007). Il-6 signaling in inflammatory bowel disease: pathophysiological role and clinical relevance. Inflamm Bowel Dis.

[27] Grieshaber-Bouyer R, Radtke FA, Cunin P, Stifano G, Levescot A, Vijaykumar B, Nelson-Maney N, Blaustein RB, Monach PA, Nigrovic PA (2021). The neutrotime transcriptional signature defines a single continuum of neutrophils across biological compartments. Nat Commun.

[28] Kolaczkowska E, Kubes P (2013). Neutrophil recruitment and function in health and inflammation. Nat Rev Immunol.

[29] Silva LM, Doyle AD, Greenwell-Wild T, Dutzan N, Tran CL, Abusleme L, Juang LJ, Leung J, Chun EM, Lum AG (2021). Fibrin is a critical regulator of neutrophil effector function at the oral mucosal barrier. Science.

[30] Dufour A, Sampson NS, Li J, Kuscu C, Rizzo RC, Deleon JL, Zhi J, Jaber N, Liu E, Zucker S (2011). Small-molecule anticancer compounds selectively target the hemopexin domain of matrix metalloproteinase-9. Cancer Res.

[31] Tanzi RE. The genetics of Alzheimer disease. Cold Spring Harb Perspect Med. 2012;2(10). 10.1101/cshperspect.a006296 PMC3475404 23028126

[32] Wendeln AC, Degenhardt K, Kaurani L, Gertig M, Ulas T, Jain G, Wagner J, Häsler LM, Wild K, Skodras A, et al. Innate immune memory in the brain shapes neurological disease hallmarks. Nature. 2018;556(7701):332–8. 10.1038/s41586-018-0023-4 PMC6038912 29643512

[33] Kawahara K, Suenobu M, Yoshida A, Koga K, Hyodo A, Ohtsuka H, Kuniyasu A, Tamamaki N, Sugimoto Y, Nakayama H. Intracerebral microinjection of interleukin-4/interleukin-13 reduces β-amyloid accumulation in the ipsilateral side and improves cognitive deficits in young amyloid precursor protein 23 mice. Neuroscience. 2012;207:243–60. 10.1016/j.neuroscience.2012.01.049 22342341

[34] Tang RH, Qi RQ, Liu HY. Interleukin-4 affects microglial autophagic flux. Neural Regen Res. 2019;14(9):1594–602. 10.4103/1673-5374.255975 PMC6557092 31089059

[35] Park J, Baik SH, Mook-Jung I, Irimia D, Cho H (2019). Mimicry of central-peripheral immunity in Alzheimer's disease and discovery of neurodegenerative roles in neutrophil. Front Immunol.

[36] Fleming TJ, Fleming ML, Malek TR (1993). Selective expression of Ly-6G on myeloid lineage cells in mouse bone marrow. RB6-8C5 mAb to granulocyte-differentiation antigen (Gr-1) detects members of the Ly-6 family. J Immunol.

[37] Cruz Hernández JC, Bracko O, Kersbergen CJ, Muse V, Haft-Javaherian M, Berg M, Park L, Vinarcsik LK, Ivasyk I, Rivera DA (2019). Neutrophil adhesion in brain capillaries reduces cortical blood flow and impairs memory function in Alzheimer's disease mouse models. Nat Neurosci.

[38] Py NA, Bonnet AE, Bernard A, Marchalant Y, Charrat E, Checler F, Khrestchatisky M, Baranger K, Rivera S (2014). Differential spatio-temporal regulation of MMPs in the 5xFAD mouse model of Alzheimer's disease: evidence for a pro-amyloidogenic role of MT1-MMP. Front Aging Neurosci.

[39] Yan P, Hu X, Song H, Yin K, Bateman RJ, Cirrito JR, Xiao Q, Hsu FF, Turk JW, Xu J (2006). Matrix metalloproteinase-9 degrades amyloid-beta fibrils in vitro and compact plaques in situ. J Biol Chem.

[40] Yin KJ, Cirrito JR, Yan P, Hu X, Xiao Q, Pan X, Bateman R, Song H, Hsu FF, Turk J (2006). Matrix metalloproteinases expressed by astrocytes mediate extracellular amyloid-beta peptide catabolism. J Neurosci.

