# Supplementary figures and images for: Increased neutrophils in inflammatory bowel disease accelerate the accumulation of amyloid plaques in the mouse model of Alzheimer’s disease
Source: Inflamm Regen. 2023 Mar 15;43:20. doi: 10.1186/s41232-023-00257-7 (PMC10015716; doi:10.1186/s41232-023-00257-7)

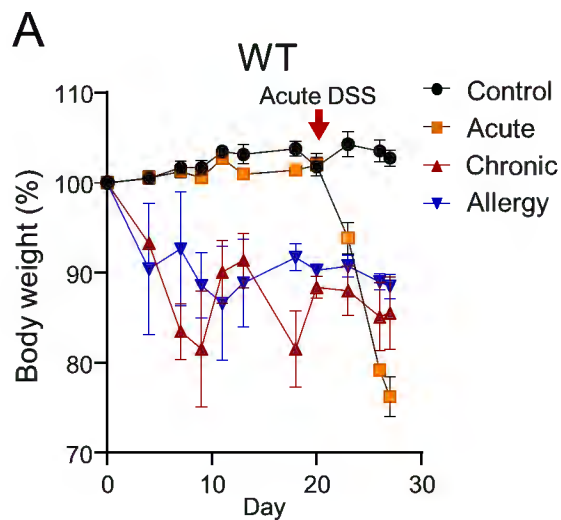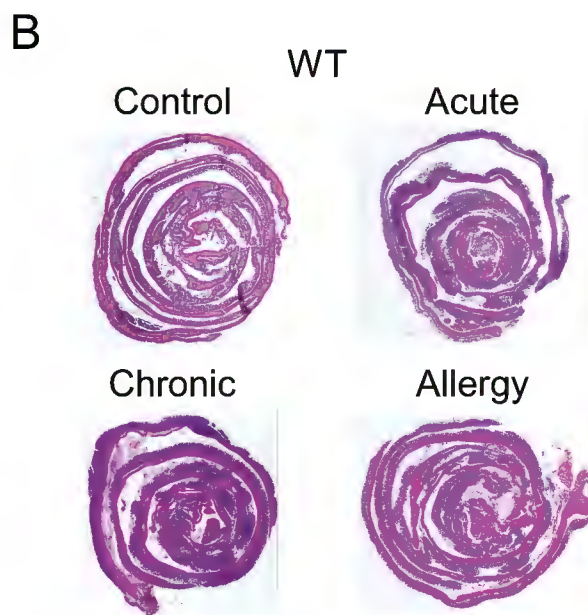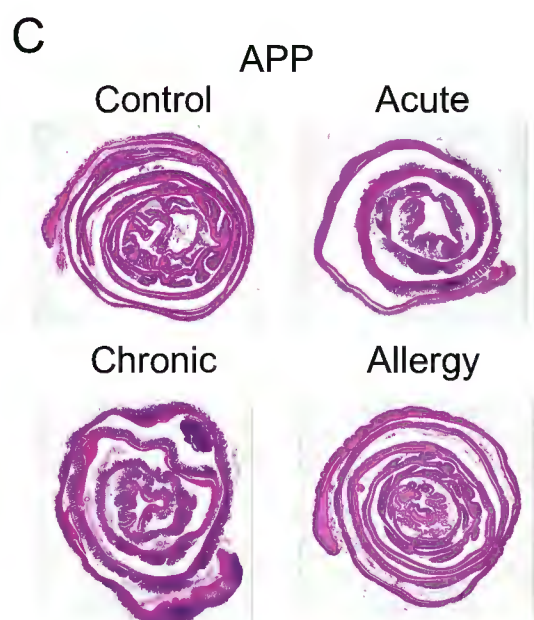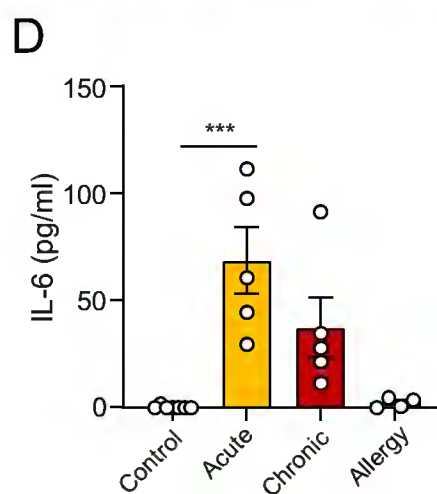

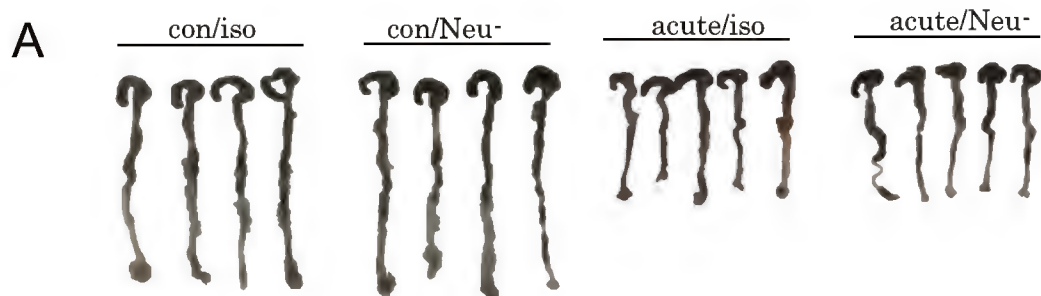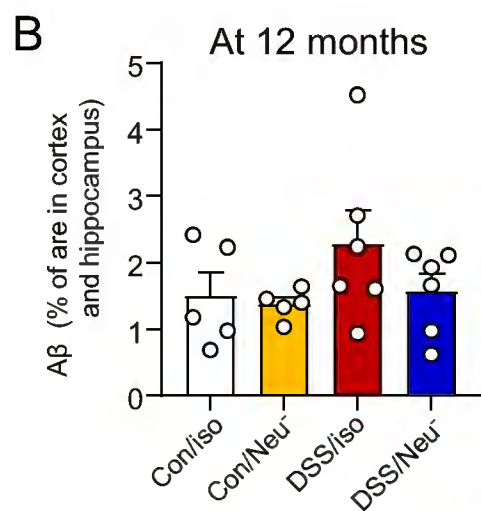

Figure. S2

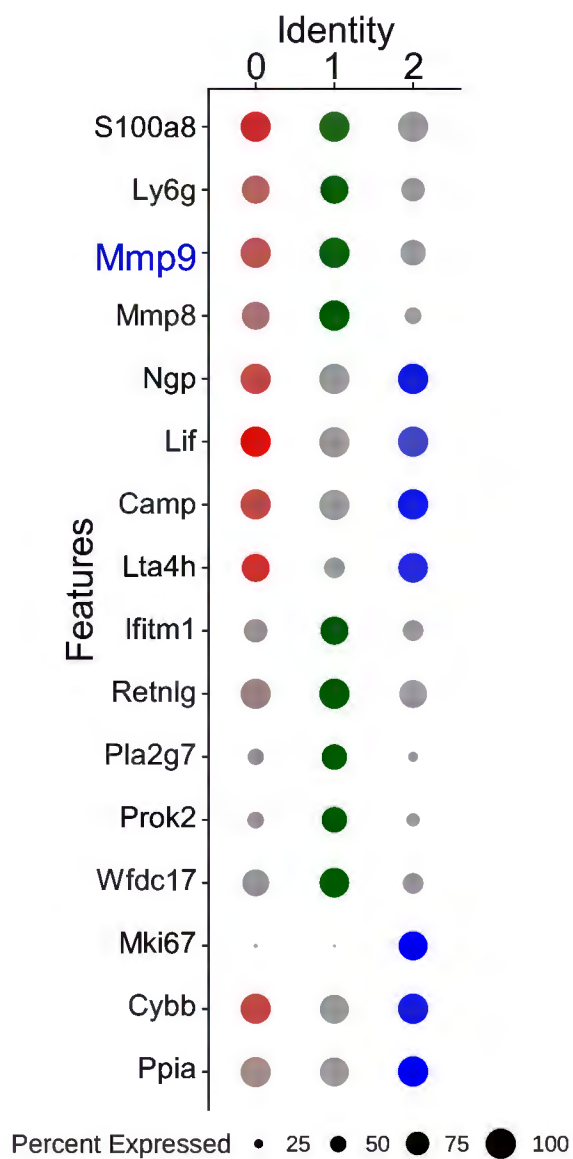

Figure. S3

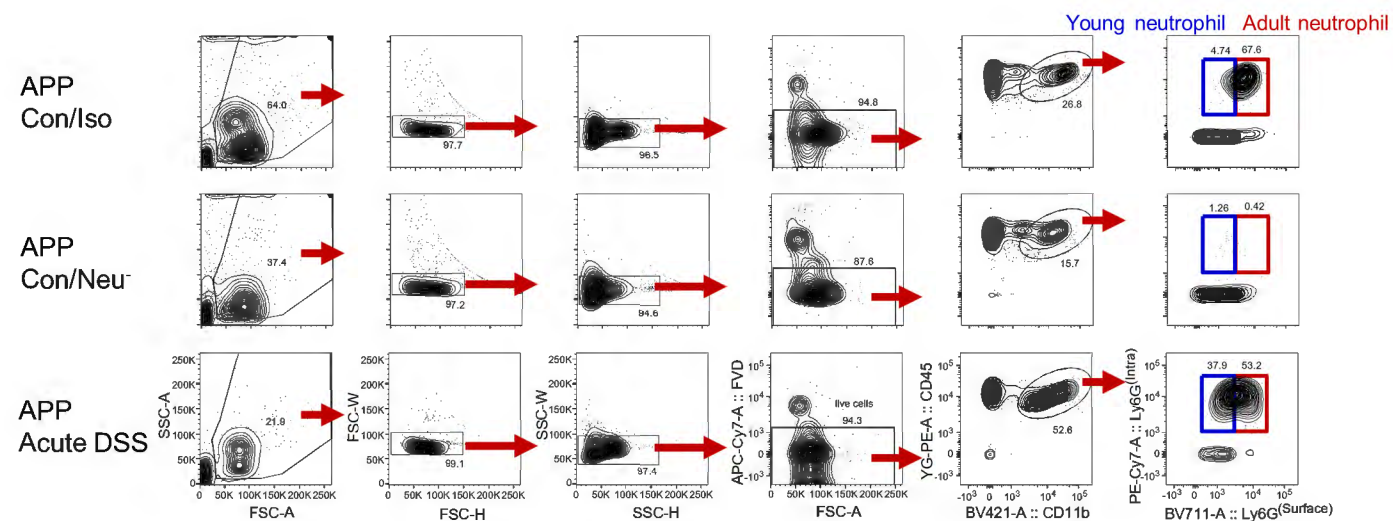

Figure. S4

Supplement: Supplementary file 1 — Additional file 1: Supplementary Figure S1. The confirmation of induction of colitis. A Body weight of colitis-induced WT mice. In acute colitis model, DSS treatment was started on day 20, indicated by the red arrow. B, C H&E staining of colon from WT (B) or AppNL-G-F mice (C) induced acute DSS colitis, chronic DSS colitis, and food allergies. D The protein levels of IL-6 in the serum of AppNL-G-F mice with colitis. Data points are individual mice from one representative of two independent experiments (A, D). P values were determined by one-way ANOVA. Data are shown as the mean ± s.e.m. Supplementary Figure S2. Reduction of Aβ plaques in the brains of AppNL-G-F mice with acute colitis by neutrophil depletion. AppNL-G-F mice drinking with fresh water or 2% DSS water were injected with anti-Ly6G antibody plus anti-rat kappa light chain antibody or isotype control antibody. A The photos of colon of AppNL-G-F mice on day 7. B The percentage of Aβ plaques area in cortex and hippocampus by immunohistochemical staining. Data points are individual mice in one experiment. Supplementary Fig. S3. Cluster-specific gene expression in neutrophils. Dot plots for selected differential expressed genes (DEG) and cell-type specific markers for the indicated clusters. Supplementary Figure S4. Gating strategy for neutrophils in the blood. Gating strategy to analyze young and adult neutrophils in the blood by flow cytometry analysis as shown in Fig. 3 H-I, and Fig. 4B. [file 41232_2023_257_MOESM1_ESM.pdf]
